# Trephining: Its Indications, Technique, After-treatment, and Sequelæ

**Published:** 1899-05

**Authors:** L. A. Merillat

**Affiliations:** Secretary of M’killip Veterinary College, Chicago


### DEPARTMENT OF SURGERY.

By L. A. Merillat, V.S.,
SECRETARY OF M'KILLIP VETERINARY COLLEGE, CHICAGO.

WITH THE COLLABORATION OF

W. E. A. Wyman, M.D.V., V.S., MILWAUKEE, WIS.

J. A. Sloan, B.Sc., M.D.V., SAN FRANCISCO, CAL.

AND OTHERS.

### Trephining: Its Indications, Technique, After-treatment, and Sequelæ.

Indications. 1. *Secondary Chronic Catarrh of the Sinuses and Nasal Cavities.* This is by far the most common indication for trephining in veterinary surgery. It is most frequently secondary to decayed molars, but may result from glanders, tumors, parasites, traumata of the skull, etc. The sinuses are often overflowing with pus or contain desiccated products which perpetuate the morbid process even after the primary cause has been removed. The treatment consists of three steps: 1. Removal of the cause, such as teeth, tumors, etc. 2. Removal of the effect—*i. e.*, pus, desiccated products, necrotic bone, etc. 3. Topical medication of the catarrhal mucosæ. These steps can only be thoroughly and energetically executed through suitable openings in the skull.

2. *Offending Teeth.* When from any cause a decayed molar cannot be removed in the usual manner, the skull immediately over its fang is trephined and the tooth driven into the mouth with a punch. All the molars can be removed in this manner except the sixth superior, whose fang lies beneath the orbit. The fifth and sixth inferior, particularly the latter, owing to the depth of the masseter muscle, are, however, reached with some difficulty. The first superior has a very delicate plate separating it from the nasal cavity, and the skull above it is so perpendicular that punching offers a rather poor method of removal.

3. *Osteomata of the Skull and Odontomata of the Molar Fangs.* The former occur at any part of the skull and in animals of all ages, while the latter occur chiefly on the fangs of the first, second, and third superior molars in horses between the ages of one and six years. They are caused by upward growth of these teeth, by the introduction of food into an open infundibulum, or by traumata. They may yield to mercurial epispastics, but trephining the apex of the tumefaction will more rapidly remove them. When they are caused by the passage of food into the tooth's infundibulum extraction is necessary, but otherwise the tooth should be unmolested.

4. *Periostitis of the diastema* is a common affection of spirited carriage, fast driving and saddle-horses. It is caused by severe bitting. Usually the inflammation terminates by the exfoliation of a small sequestrum, but in some instances the periostitis becomes an extensive ostitis, involving a considerable portion of the anterior part of the maxilla. Then trephining of the external plate becomes necessary in order to remove the necrotic segments within.

5. *Nasal tumors* are usually pedunculated, and often they reach a size sufficient to obstruct the nasal cavity on the affected side, or even diminish the lumen of the opposite chamber, by forcing the septum over from its normal position. The base of these tumors can seldom be located, and, therefore, ablation can only be accomplished through large openings in the nasal bones.

6. *Fractures of the Skull.* Fractures of the cranial bones, with displacement, are comparatively rare in the horse, and when they do occur surgical treatment is seldom successful. Not so, however, with the more exposed facial region, which is a very common seat of serious fractures. The proper treatment consists of refitting the displaced segments by pressure from within. This, of course, can only be effected by trephining the skull near the injury so as to admit the finger or instruments.

7. *Epilepsy.* This neurosis when caused by a circumscribed abnormality of the skull, meninges, or brain, is an indication for trephining. The diagnosis must, however, be confirmed beyond the possibility of a doubt before such an operation is undertaken. Indiscriminate trephining for epilepsy is no longer regarded as a rational procedure in human surgery.

8. *Turnsickness of Sheep.* The larvæ of the tænia cœnurus, a helminth of carnivora, develops within the cranial cavity of sheep and produces this fatal disease. The horse and ox are susceptible, but less so than sheep. The vesicles within which the larva develops is located by the movements of the patient, then the skull over it is trephined to allow the parasite to escape. The percentage of recoveries will vary with the number and the location of the cysts as well as the skill displayed by the operator.

9. *Abscesses in the Medullary Canal of Long Bones.* These abscesses are always traumatic in origin (in the horse), and are usually complicated with fracture at the seat of injury. Pyogenic organisms thus gain access to the marrow. The treatment consists of trephining the medullary canal at its distal extremity to permit free escape of the pus. The tibia, radius, and humerus are the bones usually affected.

L. A. Merillat.

### Surgical Items.

The young man who is beginning a surgical career, or the practitioner who is only called upon to operate occasionally, finds that a lack of knowledge in the minor points in surgical technique is the greatest difficulty which lies in his way. This department aims to furnish such knowledge, and in doing so its contributors apologize to the ultra-skilful, scientific, and experienced readers for allotting space to minor and seemingly unimportant details.—L. A. M.

The fact that Prof. Dr. Bayer's operation for cartilaginous quittor has not been universally adopted in this country, should not be regarded as a reflection on its real value, for it is admitted on all sides that no method yet advocated for these troublesome affections is more rational. It is the time, extreme care, the expense, and the grave results following the slightest error in antiseptic precautions that makes the American veterinarian undertake this operation so reluctantly.—*Id.*

